# Oxidative Stress and Inflammation in Cardiovascular Diseases

**DOI:** 10.3390/antiox10020171

**Published:** 2021-01-25

**Authors:** Raelene J. Pickering

**Affiliations:** Department of Diabetes, Central Clinical School, Monash University, Melbourne, VIC 3800, Australia; Raelene.Pickering@monash.edu

Cardiovascular diseases (CVD), which include a number of cardiac and vascular conditions, resulted in approximately 17.8 million deaths in 2017 [1]. Oxidative stress and inflammation are intricately linked mechanisms and are significant drivers in the development and progression of cardiovascular disease. In this Special Issue, the authors demonstrate the number of organs and processes affected in cardiovascular diseases. This is conclusively demonstrated in an extensive and detailed review undertaken by Podkowinska and Formanowicz that encompasses ROS, inflammation and CVD in chronic kidney disease (CKD). This review examined how oxidative stress and inflammation lead to the progression of CKD to end-stage renal disease (ESRD) and how complications of CKD accelerate CVD [2]. Another review by Ryze et al. focused on the risk of CVD in ESRD patients requiring dialysis, a process which enhances oxidative stress [3]. Together, these articles provide a great in-depth analysis of the field to date.

The original research published in this Special Issue focused on specific aspects of CVD that are just as diverse as the contents of the reviews mentioned above.

The renin–angiotensin–aldosterone system is important in regulating blood pressure (BP), where dysregulation can not only lead to aberrations in BP but also increased ROS and inflammation. Aryal et al. showed that the intrarenal AngII activation and subsequent induction of ROS could be mechanistically responsible for increased BP during chronic metabolic acidosis [4]. In another article, the treatment of heart failure in rats was considered by Pop et al., where alpha-lipoic acid was shown to have protective effects on the cardiovascular system through preventing body weight gain and by decreasing metabolic and cardiac perturbations [5].

Arellano-Buendia et al. were interested in oxidative stress in vascular complications and ESRD, since many patients, despite taking the current treatments for diabetic nephropathy, progress to ESRD [6]. Here, they looked at the ability of allicin to delay the progression of diabetic nephropathy and found that allicin had antioxidant, antifibrotic and antidiabetic properties. Other antioxidants from waste material generated from Crocus sativus petals were examined by Zeka et al. for their effects on the cardiovascular system. Crocin and kaempferol from saffron petal extract were shown to increase cell viability and decrease intracellular ROS formation [7]. Methylglyoxal (MGO), a reactive glucose metabolite that generates ROS and cellular damage, has been implicated in cardiovascular diseases. Do et al. tested 20 different compounds extracted from Peucedanum japonicum roots and determined their ability to prevent MGO-mediated endothelial dysfunction [8]. Here, they showed that isosamidin was able to prevent MGO-induced ROS and apoptotic signalling in endothelial cells and therefore suggested that isosamidin may protect against endothelial dysfunction.

Others looked at CVD in patients. Firstly, Ismaeel et al. examined the role of biopterins and oxidative stress in peripheral artery disease (PAD), where they tested molecules involved in nitric oxide (NO) bioavailability [9]. Here, they found that NO bioavailability is reduced in patients with PAD which may be caused by increased oxidative stress and reductions in tetrahydrobiopterin (BH4) [9]. Interesting data are reported by Rodriguez-Sanchez et al., where they studied the cardiovascular risk (CVR) in young subjects with or without stable coronary artery disease (SCAD) by examining the association of oxLDL and NT-proBNP. Here, they demonstrated that these markers were significantly higher in individuals with high CVR and lower in individuals with SCAD [10].

I would like to acknowledge the authors that have contributed to the Special Issue “Oxidative Stress and Inflammation in Cardiovascular Diseases”. This Special Issue has highlighted the need for further research into the mechanisms involved in CVD as well as the need for the development of novel therapeutics to treat the broad range of illnesses under the umbrella of CVD.
